# Arithmetic Memory Is Modality Specific

**DOI:** 10.1371/journal.pone.0145614

**Published:** 2015-12-30

**Authors:** Timothy Myers, Dénes Szücs

**Affiliations:** Centre for Neuroscience in Education, Department of Psychology, University of Cambridge, Cambridge, United Kingdom; University of Leuven, BELGIUM

## Abstract

In regards to numerical cognition and working memory, it is an open question as to whether numbers are stored into and retrieved from a central abstract representation or from separate notation-specific representations. This study seeks to help answer this by utilizing the *numeral modality effect* (NME) in three experiments to explore how numbers are processed by the human brain. The participants were presented with numbers (1–9) as either Arabic digits or written number words (Arabic digits and dot matrices in Experiment 2) at the first (S1) and second (S2) stimuli. The participant’s task was to add the first two stimuli together and verify whether the answer (S3), presented simultaneously with S2, was correct. We hypothesized that if reaction time (RT) at S2/S3 depends on the modality of S1 then numbers are retrieved from modality specific memory stores. Indeed, RT depended on the modality of S1 whenever S2 was an Arabic digit which argues against the concept of numbers being stored and retrieved from a central, abstract representation.

## Introduction

*“What are numbers*? *What is the nature of arithmetical truth*?*” ~ Friedrich Ludwig Gottlob Frege*

When a number is perceived, how does the human brain process it? Does it store it in the same modality in which it was presented, as in a digit or a number word? Or does the brain translate the number from diverse surface forms into a central, abstract representation of magnitude? Several number cognition models have emerged over the past two decades which differ on this point.

### 1.1. Abstract modular model

Michael McCloskey has led the way in advancing the idea that numbers are stored as a central, abstract representation. Based upon the analysis of studies with acalculic patients, McCloskey and his colleagues reported several dissociable attributes in regards to how the brain processes numbers and performs calculations. From these dissociations, they developed a framework for mathematical cognition [1, 2, 3]. One of the observations McCloskey and colleagues made was that some brain-damaged patients, when performing calculations, could *recognize* the correct answer from several possible choices but were unable to *produce* the correct answer (i.e. say or write it) when no potential answers were shown. Other patients were able to do the converse, calculate and *produce* their own correct answers but were unable to *recognize* a correct answer from several shown. Since both types of patients were able to arrive at a correct answer in one of the conditions, it seemed that the ability to calculate was present in both. From this, they concluded that calculation procedures are dissociable from number recognition and production. A second observation made was that since number recognition could be impaired while not affecting number production, and vice versa, that number recognition and number production were dissociable from each other and were associated with two distinct systems in the brain. Taking the analyses a step further, they made the assumption that the number recognition tasks accurately measured number comprehension and in so doing they also concluded that number comprehension is dissociable from number production [1]. Another condition which was manipulated in some studies was that when subjects were asked to recognize and produce answers to math problems, the numbers were also presented in different surface forms—or modalities. The numbers varied between Arabic digits, such as “5”, and number words, such as “five”. From this data a third observation was made that some patients demonstrated a dissociation between number modalities. For example, one patient was able to perform well on a magnitude comparison task with Arabic digits but not with number words. Another patient demonstrated the converse [1]. Based upon the conclusion above that the number comprehension system is distinct and dissociable from the rest of the math system in the brain, and since some patients were able to answer correctly with at least one modality even when they couldn’t answer in the other, they concluded that number comprehension is not only a separate system but that it is amodal, in other words that it is abstract in its representation of numbers.

**Fig 1:**
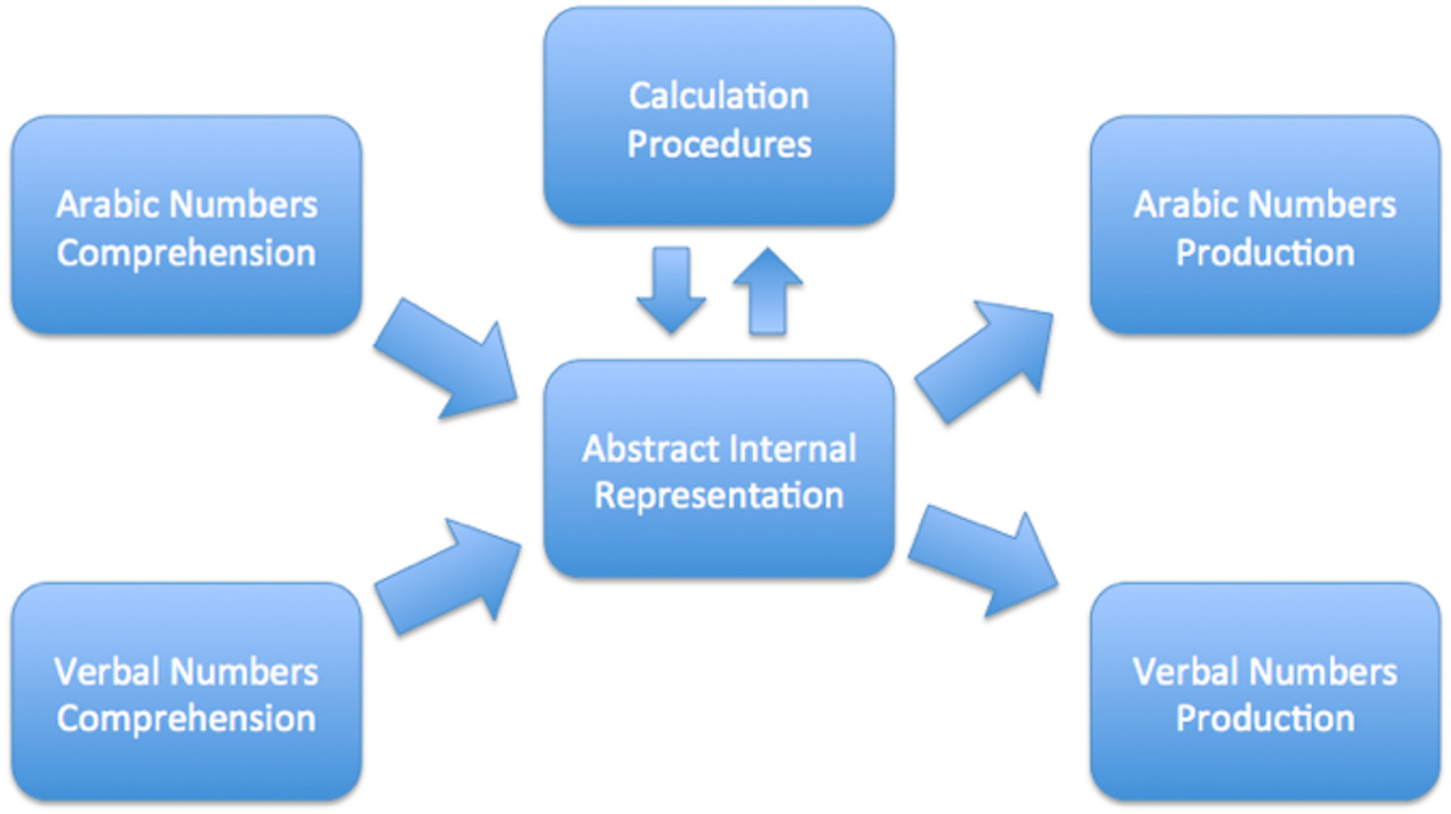
McCloskey’s Abstract Modular Model—Numbers are inputted from a specific modality such as digits or number words and are translated into an abstract representation from which they can then be outputted into a specific modality via either calculation or number production (The figure is based on Dehaene, 1992; page 28).

Using these conclusions as a foundation, McCloskey and colleagues proposed the *abstract modular* model as shown below in Fig 1. It is comprised of three distinct systems: number comprehension, calculation and number production. Central to the model is the abstract representation module which interconnects all of the systems. In the input stage of this model, the number comprehension system translates numbers from whichever modality may be encountered and encodes them into an abstract representation. From there, the abstract representations can be used to calculate or to produce numbers. The final output can be translated into whatever modality is called for.

### 1.2. Encoding-complex model

In contrast to the above framework, [4] proposed the *encoding-complex* model in which there is no assumption of a central abstract representation of number, but rather that there is a network of specific, notation-dependent number representations which can activate each other. Each representation is capable of assisting with number comprehension, number production and calculation. They proposed this model after analyzing the Arabic-number-reading-error data from McCloskey and colleagues which showed that even when a patient was not able to read a number correctly, their incorrect answers still followed a pattern. For example, if the answer was supposed to be in the “teens”, the incorrect answer which was produced or recognized would likely not be in the 0–10 range or in the 20–90 range but would also be in the “teens” [1]. Campbell and Clark conducted a regression analysis which looked at multiple factors which had a possible effect on number processing, such as odd/even agreement, numerical nearness, and visual similarity. They found that the convergence of difficulty with two or more of these factors produced a higher probability of an incorrect answer being chosen. Since two of these factors, visual and numerical similarity, were based on visual stimuli, and since these factors had an effect on the patient’s ability to produce numbers, and since in McCloskey and colleague’s model the visual stimuli should go no further than the input stage (before numbers are translated into an abstract code and well before number production), Campbell and Clark reasoned that these findings were inconsistent with the *abstract modular* model. They also found the effect caused by odd/even agreement, which in the *abstract modular* model should be isolated in the calculation system, to be inconsistent [4]. They proposed that the multiple numerical and visual stimuli, which appeared to be present when the incorrect answers were chosen, could be explained more accurately by stating that it was the convergence of the multiple stimuli that made the response difficult. In other words, due to a damaged brain area, the patient would be receiving multiple visual and numerical cues at the same time that would impair their ability to choose a response. They noted that this explanation fit well with other studies which have demonstrated similar models for other aphasic conditions [4], and they contended that the *encoding-complex* model fit the observations of their experiments more accurately than the *abstract modular* model.

### 1.3. Triple code model

**Fig 2:**
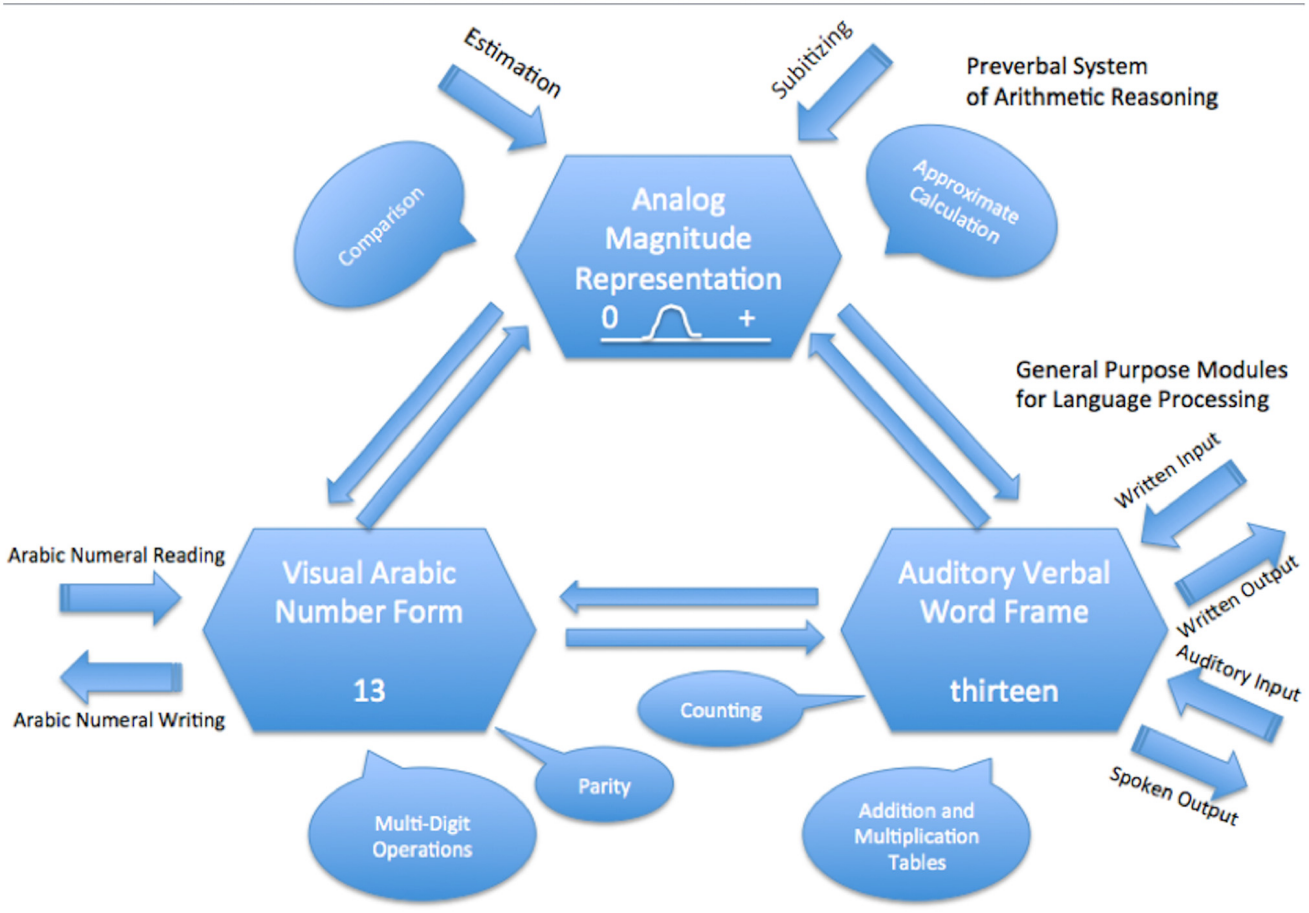
Dehaene’s Triple Code Model—Numbers are stored in three individual yet integrated codes. The Analog Magnitude code is an abstract representation that provides the basic sense that gives meaning to numbers (The figure is based on Dehaene, 1992; page 31).

Another model that is popular currently is the *triple code* model proposed by Stanislas Dehaene [5] shown in Fig 2 below. Aspects of Dehaene’s model can be seen as a combination of the above two models. It consists of three separate yet integrated neural codes. One code, located in the left and right inferior ventral occipito-temporal areas, is responsible for processing Arabic digits. Another code, located in the left perisylvian area, is responsible for processing number words as well as the algorithms used for calculating and the math facts that are stored in memory. Both of these codes are modality and notation-dependent, similarly to Campbell and Clark’s *encoding-complex* model. The third code in Dehaene’s model, located in the left and right intraparietal sulci, especially in the horizontal intraparietal sulci (hIPS), is responsible for abstract, analogue representations of number. This is the code which allows for making comparisons and for comprehending magnitude. Also similarly to the *encoding-complex* model, Dehaene’s three codes are interconnected and can be called upon depending on the task. In regards to the semantic representation of number, Dehaene has proposed that the magnitude representation area, hIPS, is the module where abstract representations of numbers are encoded. His model provides for a direct path between the Arabic and verbal codes which bypasses the magnitude code; however, this is only in instances where the conscious meaning of number is not required. For all number processing and calculations in which the meaning of number must be comprehended, the numbers must be encoded into the same abstract representation area (i.e. magnitude area) irrespective of the modality in which the number was first presented [6, 7, 8]. In this sense, the *triple code* model is very similar to McCloskey’s *abstract modular* model in that there is a central abstract area for the representation of number which acts as a bottleneck for number cognition.

It is to note that Campbell and Epp [9] proposed a numerical cognition model which combined aspects of the *encoding-complex* and *triple code* models. Their revised model assumed modular numerical representation, so concerning the question of whether numbers are stored as notation-dependent or as amodal abstract representations, this encoding complex version of the *triple code* model would hold the same prediction as the *encoding-complex* model: that is a prediction for notation-dependent numerical representations.

### 1.4. Evidence for central abstract representation being the default representation

Many in the math cognition field lean toward the idea of a central abstract representation of number being the default representation with which numbers are processed in the human brain. In addition to the lesion studies described above by McCloskey and colleagues, further evidence in support of this view is summarized below.

One stream of evidence which supports the central abstract representation of number stems from observing effects which are independent of the presented modality of numbers. In their 2009 paper [10], Cohen Kadosh and Walsh summarized several of these studies. One example of this is the numerical distance effect (i.e. subjects have a faster RT as the numerical distance between two numbers decreases). Several studies have shown that the numerical distance effect is demonstrated in the same way irrespective of the modality in which the numbers were presented. Another example is the SNARC effect (i.e. subjects have a faster RT when responding with their right hand for numbers on the right side of the mental number line, and a faster RT with their left hand for numbers on the left side of the mental number line), which has also been shown to be independent of the modality of the presented numbers. Also, in a study with children, Thevenot and Barrouillet [11] reported that, in line with predictions of the triple code model, encoding times were longer for numbers to be used in addition and subtraction than for numbers to be used for comparison.

Another stream of evidence for the central abstract representation of number comes from functional magnetic resonance imaging (fMRI) studies. First, the hIPS area of the brain has been shown to be more activated by tasks in which calculations were done with numbers or when comparing numbers than when numbers were simply viewed and recognized [12, 13]. From this it has been concluded that, since calculation and comparison utilize a quantitative representation of numbers more so than does the recognition of numbers, a greater activation in the hIPS which correlates with those tasks may suggest that the hIPS is responsible for abstract, quantity representations [14]. Second, the hIPS area has also been shown to have greater fMRI activation for tasks that require approximation rather than tasks that require precise calculation even when the difficulty of the task is controlled for [12, 14, 15, 16]. This also leads to the conclusion that the hIPS processes abstract quantity. Third, the hIPS is activated regardless of the modality in which numbers are presented [13, 17]. In their 2003 paper [14], Dehaene and colleagues reviewed several fMRI studies in order to examine a three-dimensional intersection of the brain which was activated during various numerical tasks. They found that the hIPS was the one area that was activated during all of the numerical tasks (i.e. during digits, number words and non-symbolic dot patterns). If the hIPS is responsible for processing abstract quantity, and if the presented modality of numbers does not affect its activation, an argument can be made that the hIPS is acting as an area for abstract numerical representation that is both separate and amodal. Considered together, the observed effects which are independent of number modality, as well as the evidence for the activation of one brain area which is activated in both symbolic and non-symbolic number tasks, seems to lead to the conclusion that number representation is abstract and may be centrally located in the hIPS.

### 1.5. Evidence against a central abstract representation being the default representation

Although the idea of a central abstract representation area remains popular, there are also critics of it. Some of the arguments against abstract representation being the default representation for numbers stem from perceived confounds in the studies which support it. As an example, McCloskey and colleagues [1], based the logic which led to their *abstract modular* model on the conclusion that number comprehension is dissociable from number production and calculation. However, a close inspection reveals that the actual task used by McCloskey and colleagues to measure number comprehension was a number *recognition* task. The assumption that number recognition tasks measure number comprehension may be an overreach. Factors which are further upstream than number comprehension could be causing this number recognition impairment. It seems more likely that poor performance in the recognition task could simply signify an impaired ability to encode visually presented numbers.

In regards to the evidence from effects which are independent of the presented modality of numbers, several difficulties with the conclusions are noted. First, as Cohen Kadosh and Walsh [10] pointed out, the majority of evidence in favor of abstract coding is based upon null results (i.e. the absence of a difference between number modality and the behavioral output or blood oxygenated level dependent [BOLD] response). The observations of the null results may simply be due to not having enough statistical power or not having sensitive enough paradigms. Second, the fMRI evidence summarized above assumes that because the same area is being activated, the same representation of number is active. However, such data may also be the consequence of activating a comparison process common to several representations rather than activating a core representation [18]. Third, several adaptation designs relied on the crucial assumption that participants were not paying conscious attention to the numerical content of stimuli. However, this assumption was probably violated in several studies [18]. Fourth, fMRI resolution in several studies may not have been precise enough to ascertain whether it was one brain process or several which were occurring in a given activated area [10, 18, 19, 20]. Fifth, several studies attempting to measure an abstract number representation may have had serious visual stimulus property confounds [21, 22, 23].

Another stream of evidence against a central abstract representation being the default representation for numbers in the human brain is derived from studies which *have* observed significant differences which are dependent on number modality. Cohen Kadosh and Walsh [10] summarize several such studies in which participants are presented with differing surface forms of numbers. One study utilized the size congruity paradigm in which subjects compared numbers according to physical size and attempted to ignore numerical magnitude. When incongruent trials have slower RTs than congruent trials, it signifies that automatic processing of numbers is occurring. In this study, participants did not demonstrate automaticity of number processing with Kana script (Japanese number words), but they did with Kanji (Japanese digits) [24]. Another study had participants compare digits and number words by comparing the effects of numbers in the previous trial to the current one. When there was a short response-to-stimulus-interval they observed an interaction between different number modalities and the numerical distance in reaction time, between modalities and modality repetition and error rates, and between modality and the magnitude distance between the number in the previous trial to the number in the current trial. They noted that these observations argue for a non-abstract representation of number [25]. In yet another study, the participants decided whether two simultaneously presented numbers were the same or different. In one condition digits as well as number words were used in a mixed, random manner. When the participants based their decision on physical differences as opposed to numerical magnitude, a distance effect was observed which was independent of number modality [26].

Another interesting surface form study was conducted in 2004 by Campbell, Parker, and Doetzel [27]. In experiment 1 of their study, participants were presented with simple addition, multiplication, and parity tasks (the numbers were either Arabic digits or written number words). As well as generally finding that written number words are correlated with slower RTs and worse accuracy (which is consistent with studies utilizing surface form), they also found a parity effect in which participant’s performance decreased (RT and accuracy) when adding odd numbers more so than with even and which decreased further when written number words were shown as contrasted with Arabic digits. As this parity effect was only observed for the simple addition tasks, not with multiplication, it was concluded that the performance costs were not caused in the encoding stage but rather that surface form affected calculation processes. This argues against a central abstract representation model as in such a model the original surface form of numbers should not affect processes after the encoding stage. In experiment 2 as well as in a previous study [28], participants reported the strategy they utilized after each trial of a simple addition/parity task (2004) or simple addition task (2001). In both, procedural strategies were reported much more often with written number words while recall strategies were used more so with Arabic digits. The results of these experiments show that surface form affects central processing of cognitive arithmetic and would not be expected if numbers were converted from their original surface form into a central, abstract, amodal representation.

Although not a surface form study, Thevenot and Barrouillet [29] observed an effect cogent to this discussion. They conducted experiments in which participants were asked to either add, subtract or compare two successively presented numbers. After viewing the first number the participants would press a key in order to be presented with the second number. They found that participants were faster to process numbers when they were going to compare it to the second number as opposed to when they were going to add or subtract. Since this seemed to demonstrate that the participants were using difference encoding processes for comparing and calculating numbers, they concluded that this argued against a central abstract model and agreed more with an encoding complex version of the triple-code model—in other words, a version of the triple code model that does not require the magnitude representation area to be the default area for numbers to be stored.

Finally, a surface form study which provides both behavioral and neuroscience evidence for modality specificity was conducted in 2004 by Szűcs and Csépe [30]. They reported the *numeral modality effect* (NME) which showed interactions with the presentation of different number modalities. The paradigm used in this study compelled participants to retrieve a number from memory that had previously been presented as one of three different modalities (Arabic digit, written number word, or aurally heard number word). After retrieving the number from memory, the participants added it to a second number which was always an Arabic digit and then confirmed whether a third number (also always a digit) was the correct sum or not. Since the second presented number was always an Arabic digit, and since the number that was presented previously was retrieved from memory, if the first number was translated into an abstract representation then the task of adding the two numbers and verifying the answer should have produced RTs and event-related potential waveforms which were not dependent on the modality of S1. However, the behavioral and event-related potential (ERP) analysis showed an effect that was dependent of the modality of S1, between the conditions in which S1 was a written number word or an aurally heard number word, which argues for notation-dependent representations. It is to note that the RTs and ERP amplitudes of the condition in which S1 was an Arabic digit was in between the other two conditions and no significant effect was observed between them.

### 1.6. Summary of study

**Fig 3:**
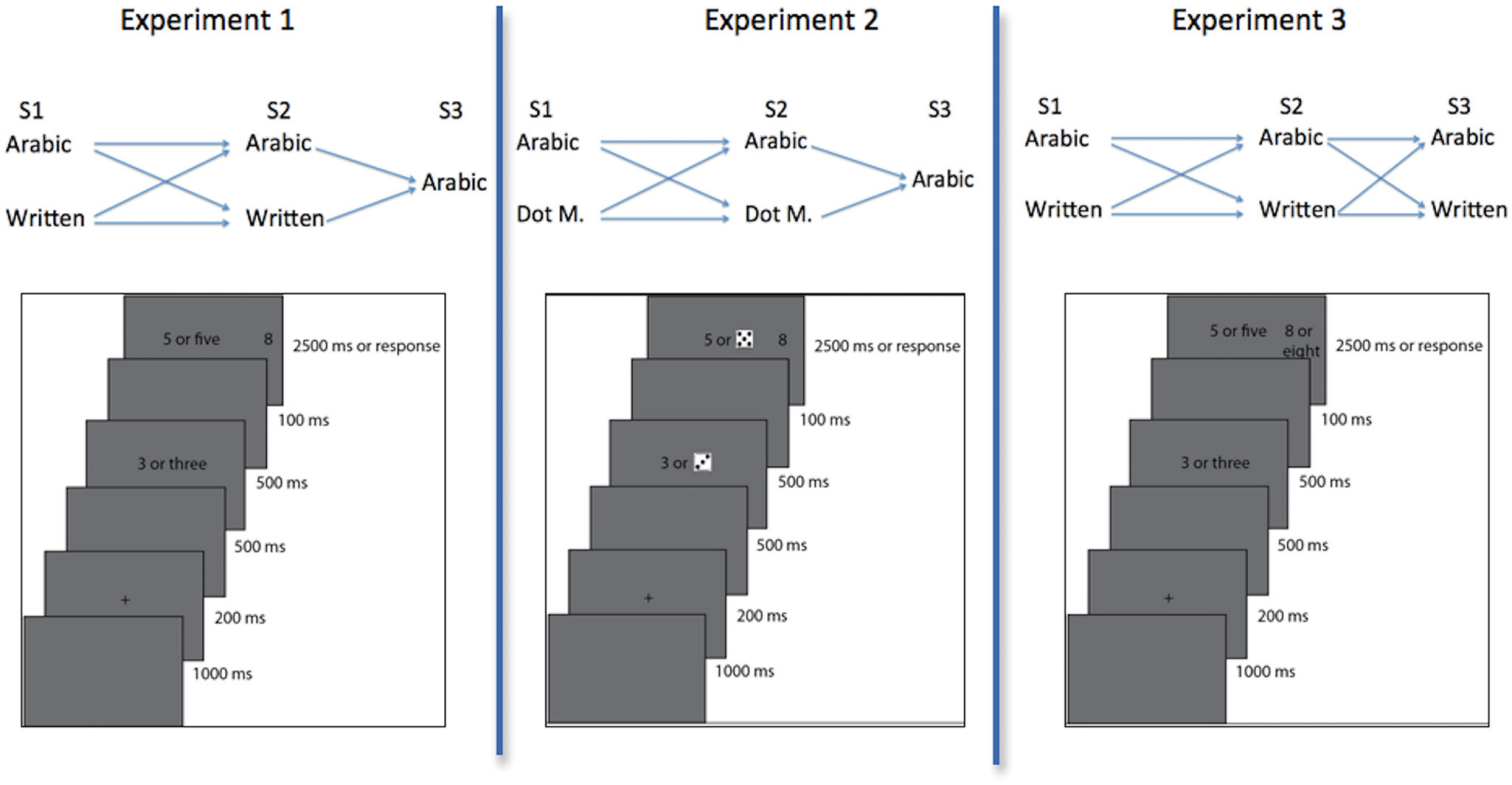
Design of experiments. Trials started with a fixation cross. In experiment 1 the participants were presented with numbers 1–9 as either an Arabic digit or a written number word at S1 and S2 and then as an Arabic digit at S3. S2 and S3 were shown simultaneously with the later being shown to the right of the screen. The participants were to remember S1, add it to S2 and then verify whether S3 was the correct sum. In experiment 2, the same paradigm was employed as in experiment 1 using Arabic digits and dot matrices. In experiment 3, S3 was also presented as either an Arabic digit or a written number word.

Our study sought to provide additional evidence concerning whether number storage and retrieval relies on notation specific processes. We utilized the *NME* paradigm introduced by Szűcs and Csépe [30] in three experiments. As shown in Fig 3, in all experiments participants were presented with a single-digit number (1–9) at the first (S1) and second (S2) stimuli. S1 was presented briefly and after disappearing was followed by S2 and a third (S3) stimulus shown simultaneously. Participants were instructed to add S1 and S2 and then verify whether the number at S3 was the correct sum or not. In the present series of studies we used 2x2x1 and 2x2x2 factorial designs instead of the 3x1x1 design employed by Szűcs and Csépe [30]. The paradigm was changed in this way in order to control for the possibility that direct encoding into the Arabic digit modality was being facilitated, either explicitly or implicitly, by S2 always being an Arabic digit. In order to further generalize findings we also fully randomized the trial order (S1xS2 modality) while the original study used a blocked design of S1 modality.

In Experiment 1 (2x2x1 design) the numbers at both S1 and S2 varied randomly in regards to their presentation modality. We utilized the Arabic digit (e.g. “8”) and written number word (e.g. “eight”) modalities in order to observe whether the *NME* could be replicated with them. In the previous experiment [30], the effect was only observed between the written number word and aurally heard number word conditions. We expected that the modality (Arabic digit or written number word) of S1 would affect the processing time of S2/S3. That is, the main dependent variable was the RT measured at S2/S3. We argued that when S2 and S3 appeared, participants had to evaluate S2 (i.e. perceptually process it and access its meaning), add S1 to S2, and then decide whether S3 matched the solution. These steps obviously required that participants keep S1 in their working memory and retrieve it when necessary. Hence, we reasoned that the RT difference measured at S2/S3 would be related to the differential retrieval speed of S1 from modality specific memory stores [30]. (It is to note that a mere speed difference in function of the modality of S2/S3 is uninteresting as it probably simply reflects encoding differences in function of stimulus modality).

Experiment 2 repeated the same design using dot matrices instead of written number words to replicate the NME and to observe how dot matrices, considered to be more abstract, are processed relative to written number words and Arabic digits. Experiment 3 extended Experiment 1 by investigating what happened when S3 was also randomly presented as either a digit or a number word (2x2x2 design). This allowed for observing whether the modality of S3 (always an Arabic digit in Experiment 1) led to preferential processing of Arabic digits.

## Experiment 1

### 2.1. Hypothesis of Experiment 1

Experiment 1 replicated and extended the design of the Szűcs and Csépe [30] study. The dependent variables were RT and verification accuracy measured for solutions, that is at S2/S3. We hypothesized that if RT, measured at S2/S3, depends on the modality of S1, then numbers are being retrieved from modality specific memory stores as opposed to being retrieved from a central abstract representation area. The reasoning for this is that if numbers are stored as notation-dependent representations, then the modality of S1 is indicative of the modality of the representation that is being retrieved in order to add to the number at S2. If, on the other hand, the number from S1 is translated into a central abstract representation, then the participant would retrieve numbers from that abstract representation in order to add to the number at S2, making the original modality of S1 inconsequential in regards to RTs.

### 2.2. Methods of Experiment 1

#### 2.2.1. Ethical Approval

This study as well as the consent procedure was approved by the Ethical Committee of the Department of Psychology at the University of Cambridge. Participants provided written consent to participate after reading the information sheet and being given the opportunity to ask questions.

#### 2.2.2. Subjects

Twenty adults (ages 18–35; median age: 23) from the Cambridge, UK area participated in this study. All were students, nine of which were engaged in graduate studies and the rest were undergraduates. Twelve participants were female and all reported themselves as being unimpaired in regards to math ability.

#### 2.2.3. Stimuli and procedure

Participants sat in front of a laptop at a table in a comfortable chair. They were given instructions concerning the task and were given the opportunity to ask questions and seek clarification. After a practice trial to familiarize them with the paradigm, they participated in 4 blocks consisting of 150 trials each. They were permitted to rest for as long as needed between each block. The stimulus presentation was written in the Python language using the program *PsychoPy* [31]. During each trial, participants would be presented with a blank screen for 1000 ms after which a fixation cross would appear in the center of the screen for 200 ms. After a 500 ms pause, they would then be presented with the first stimulus (S1) for 500 ms. S1 was a randomly chosen number (1–9) that would be randomly presented as either an Arabic digit (A) or a written number word (W). After a 100 ms pause following the disappearance of S1, they would then be presented simultaneously with the second (S2) and third (S3) stimuli. S2 was also a randomly selected number (1–9) that would be randomly presented as either the A or the W condition. Just as with S1, it would be presented in the center of the screen. S3 was presented on the right side of the screen. It was always an Arabic digit and was either the correct sum of S1 and S2 or it was not, which was decided at random. It was correct about half of the time. If it was not the correct sum, it was incorrect by either +1, -1, +4, or -4 each about ¼ of the time chosen randomly. S2 and S3 would stay on the screen for 2500 ms or until the participants chose a response. If S3 was the correct sum, participants were instructed to press the “L” key. If not, they were instructed to press the “A” key. We chose to vary the Arabic digits and number words within the trial blocks.

#### 2.2.4. Analysis

The data were analyzed with a Correctness x S1 Modality x S2 Modality ANOVA. The effect size will be communicated by partial eta squared (*Partial η* ^*2*^).

### 2.3. Results of Experiment 1

#### 2.3.1. Effect of the modality of S1 and S2 on the RTs

**Fig 4:**
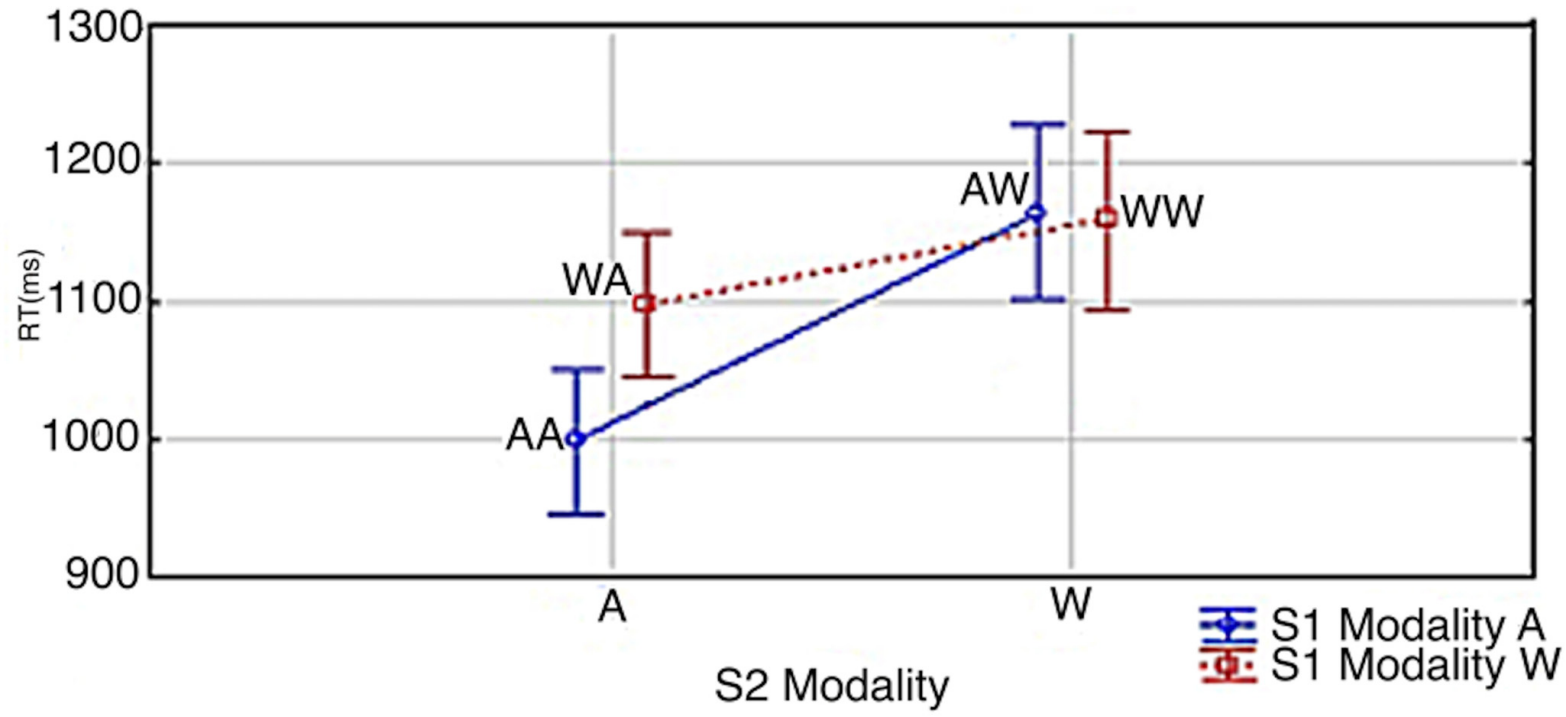
S1 Modality x S2 Modality. Vertical bars denote 0.95 confidence intervals for repeated measures ANOVA (Hollands & Jarmasz, 2009). S1 and S2 modality is signified by ‘A’ and ‘W’; i.e. Arabic Digit (A) vs. Written Number Word (W). As an example, AW signifies an Arabic digit at S1 and a written number word at S2.

RTs are shown in Fig 4. Data were assessed by a Correctness x S1 modality x S2 modality repeated-measures ANOVA. The .95 confidence intervals were computed for repeated-measures ANOVA (see Hollands and Jarmasz [32]). There was an S1 modality x S2 modality interaction (AA = 992 ms, WA = 1085 ms, WW = 1134 ms, AW = 1159 ms; S1 Modality x S2 Modality: *F*(1,19) = 34.067, *p*<0.0001; *Partial η* ^*2*^ = 0.32) which shows that there was no impact on the retrieval speed of S1 when S2 was a written number word; however, when S2 was an Arabic digit it was processed quicker when it was preceded by a digit rather than a written number word. According to S1 modality x S2 modality Tukey post hoc contrasts, there were significant differences between all conditions except between AW and WW (AW vs WW: p<0.3109; AA vs. AW: p<0.0002; AA vs. WA: p<0.0002; AA vs. WW: p<0.0002; AW vs. WA: p<0.0005; WA vs. WW: *p*<0.0162). In addition to this, the RTs were 33 ms faster in all conditions of S2 when S1 was an Arabic digit as contrasted to when it was a number word (1109 ms vs. 1076 ms; main effect of S1 Modality: *F*(1,19) = 12.833; *p*<0.001; *Partial η* ^*2*^ = 0.31). The RTs were also 108 ms faster in all conditions of S1 whenever S2 was an Arabic digit (1147 ms vs. 1039 ms; Main effect of S2 Modality: *F*(1,19) = 76.484; *p*<0.0001; *Partial η* ^*2*^ = 0.56). In regards to the effect of correctness of the presented sum at S3, The Arabic digit at S3 was responded to 65 ms faster when it was the correct sum as opposed to when it was incorrect (1060 ms vs. 1125 ms; Correctness: *F*(1,19) = 39.998, *p*<0.0001). There was no interaction between the correctness of S3 and the modalities at S1 and S2 (Correctness x S1 Modality x S2 Modality: *F*(1,19) = 2.641, *p*<0.1206).

#### 2.3.2. Effects on accuracy

Accuracy was also assessed by a Correctness x S1 modality x S2 modality ANOVA. There were no significant effects in regards to accuracy, including specifically no effects for the S1 Modality (*p* = 0.22), the S1 Modality x S2 Modality (*p* = 0.3), or the Correctness x S1 Modality x S2 Modality (*p* = 0.26) (See Table 1 for accuracy rate).

**Table 1:** Overall accuracy rate (percent correct) for all three experiments. S1, S2, and S3 (where shown) modality is signified by ‘A’ and ‘W’; i.e. Arabic Digit (A) vs. Written Number Word (W). As an example, AWW signifies an Arabic digit at S1, a written number word at S2, and a written number word at S3.

Conditions for Exp. 3	AAA	AAW	AWA	AWW	WAA	WAW	WWA	WWW
Conditions for Exps. 1,2	AA	AW	WA	WW				
Exp. 1	96%	95%	95%	95%				
Exp. 2	97%	88%	88%	87%				
Exp. 3	94%	84%	93%	83%	95%	89%	95%	89%

### 2.4. Discussion of Experiment 1

Results confirmed and extended the effect observed from Szűcs and Csépe [30]: in the trials in which S2 was an Arabic digit, when S1 was also an Arabic digit the RTs at S2/S3 were significantly faster than when S1 was a written number word. Importantly, this means that RT depended on the modality of S1. S1 had a maximum potential encoding time of 600 ms, hence there was ample time for encoding S1 and for it to be available to be retrieved from memory at the time of measuring RT. Since there should be no RT difference if S1 is always stored into and retrieved from a central representation to always be added to an Arabic digit at S2, our finding seems to indicate that the numbers from S1 were being retrieved from modality dependent representations. The results, in this condition, similarly to the results of Szűcs and Csépe [30], lead us away from the idea that numbers are stored in a central abstract representation and toward the conclusion that numbers are stored in a modality-dependent manner. It is to note that these results extend the findings from Szűcs and Csépe [30] as in that study a significant effect was only observed between the aurally heard and written number word conditions and not between Arabic digits and written number words as is shown here.

**Fig 5:**
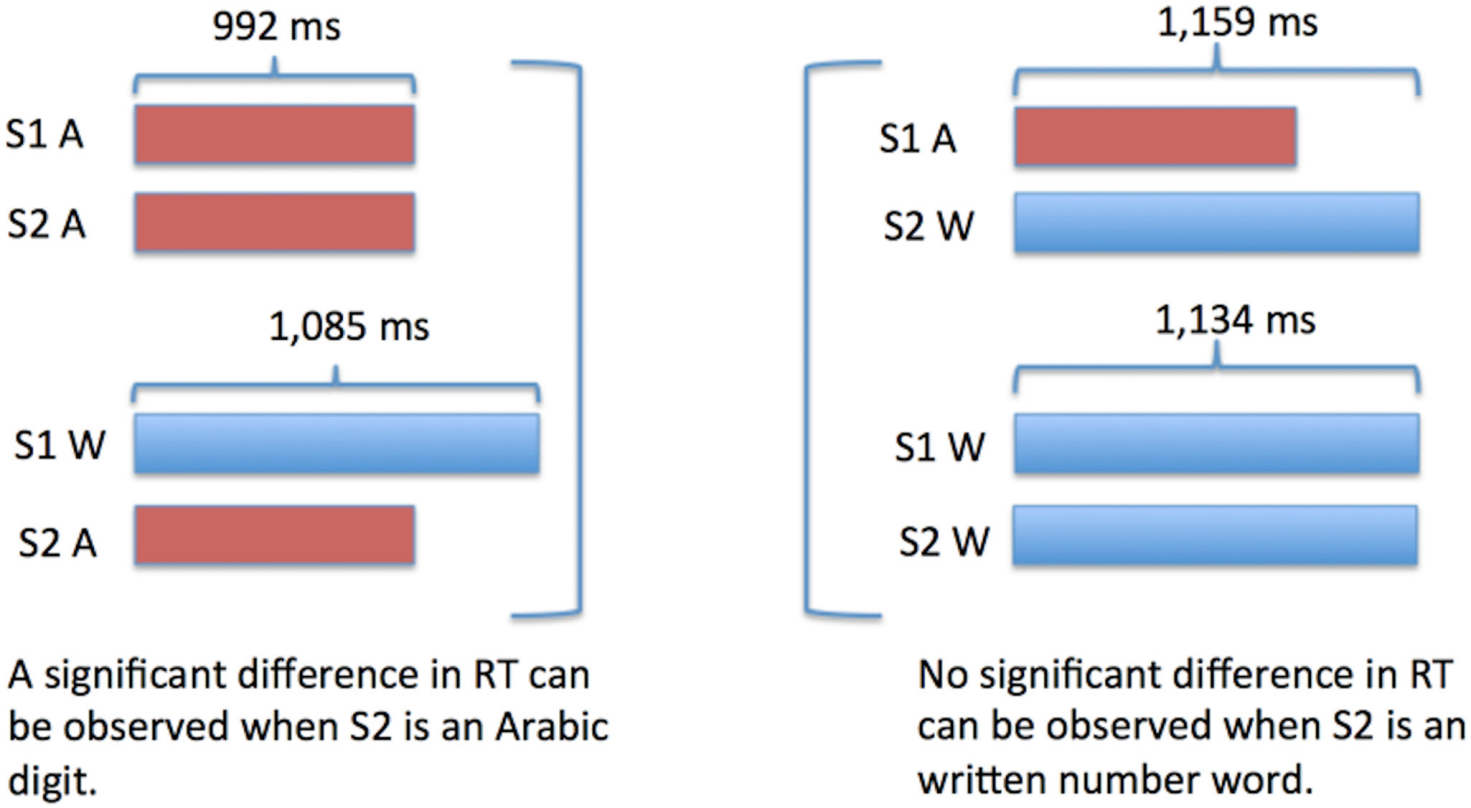
A = Arabic digit & W = written number word. This figure demonstrates how the longer encoding time of W at S2 would mask RT differences if S1 retrieval and S2 encoding occurred in parallel. In the AW condition, even though A may be retrieved more quickly, the participant would not be able to answer until W had been encoded which would make the RT observed indistinguishable from the WW condition.

We also included a condition, varied randomly, in which S2 was a written number word. This was to control for the possibility that direct encoding into the Arabic digit modality was being facilitated in previous experiments, either explicitly or implicitly, by S2 always being an Arabic digit. Irrespective of the modality of S2, the RTs (measured at S2/S3) were faster whenever S1 was an Arabic digit. This leads us to the conclusion that presenting S2 as an Arabic digit in all trials in previous experiments did not preferentially bias the participants to process digits. However, as well as confirming the validity of previous experiments, introducing this new condition also resulted in an unexpected phenomenon. In the trials in which S2 was a written number word, there was not a significant difference in RTs based on the modality of S1. This lack of significance could be interpreted as the RTs being independent of the modality of S1, which would point towards the central abstract representation hypothesis as being correct. Indeed, if this had occurred in both conditions, this would be the most reasonable conclusion. However, this did not occur in both conditions, so what can be drawn from these results which at first glance appear to contradict? First, it can be safely assumed that the two primary conditions in this experiment (S2 = Arabic digit & S2 = written number word) are not causing nor are correlated with different encoding processes at S1. In other words, participants are likely not encoding S1 into a domain specific representation when S2 will be a Arabic digit and into a central abstract representation when S2 will be a written number word. Simply put, this is because the participants could have had no reasonable idea as to which modality S2 would be presented as in any given trial as the modalities were varied randomly within blocks. Second, when interpreting results, its more reasonable to accept a condition which produces an effect, all else being relatively equal, over a condition which does not produce an effect. When an effect is not observed it does not mean that an effect does not exist; however, the converse cannot be as strongly stated for an effect that is observed. Third, in the condition in which S2 was an Arabic digit, no other explanation apart from notation-dependent encoding appears to be able to reasonably explain why the RTs at S2/S3 would be dependent on the modality of S1, assuming that 600 ms was enough time to initially encode S1. Fourth, there *is* a reasonable explanation as to why there was no effect observed in the condition in which S2 was a written number word. Taking into account that RT’s were significantly longer when S2 was a number word as compared to when it was a digit, it seems likely that the longer encoding time that it takes to interpret S2 when it is a written number word may mask significant time differences in retrieving S1 (see Fig 5). This would also suggest that the retrieval of S1 and the encoding of S2 happen in parallel rather than sequentially (see for example the perceptual flow model of Eriksen and Schultz [33]). This seems biologically most feasible given that the human brain is a heavily parallelized, rather than sequential, processing system. Hence, participants likely start to retrieve S1 whenever it becomes necessary and economical to have it in working memory (for example, at the presentation of S2). In parallel with this retrieval process, they also start to encode S2. When both S1 and S2 become available (S1 retrieved and S2 encoded) they carry out the required operation. When encoding S2 takes a short amount of time (S2 is an Arabic digit), it is possible for S1 retrieval differences to manifest in RT outcomes. In contrast, when S2 encoding takes more time (S2 is a written number word), by the time S2 is encoded, S1 had already been retrieved from both Arabic and Written modalities. Hence, retrieval speed differences in function of S1 modality only manifest when S2 encoding is relatively fast.

Another point to consider is that Arabic digits and written number word surface forms have been shown to elicit different arithmetical strategies (retrieval or calculation) from participants [27, 28]. These different strategies which are used (and not just numerical processing) can affect RTs. There are a few things to note from this. First, as Campbell et al. point out, this phenomenon actually confirms the predictions of the encoding-complex model (i.e. that numbers are stored as notation-dependent representations) as it shows that number modality affects central processing. Second, in our experiment it does not affect our hypothesis as even if different strategies are used, they can only be applied if the participants retrieve a number from different modalities. If numbers were being retrieved from a central abstract representation each trial, then presumably similar strategy choices would be elicited for each trial.

## Experiment 2

### 3.1. Hypothesis of Experiment 2

Experiment 2 operated with the same hypothesis as experiment 1, that if numbers are being retrieved from notation-dependent memory stores then RTs at S2/S3 should be dependent on S1 modality. We utilized canonical dot matrices with the Arabic digits instead of written number words. Since the triple-code and abstract modular models propose a central abstract representation area for number, we were interested in observing the participant’s RTs with dot matrices as they are considered to be more abstract than Arabic digits.

### 3.2. Methods of Experiment 2

#### 3.2.1. Subjects

Twenty participants, twelve of which were female, participated in this study. They were between the ages of 18 and 35 (median age: 23) and were from the Cambridge, UK area. Six of these also had participated in Experiment 1. Seventeen of the participants were students or former students from the University of Cambridge with the others being residents from the Cambridge area. Eleven of the students were engaged in or had completed graduate studies and the rest were undergraduates. All reported themselves as being unimpaired in regards to math ability.

#### 3.2.2. Stimuli and procedures

**Fig 6:**
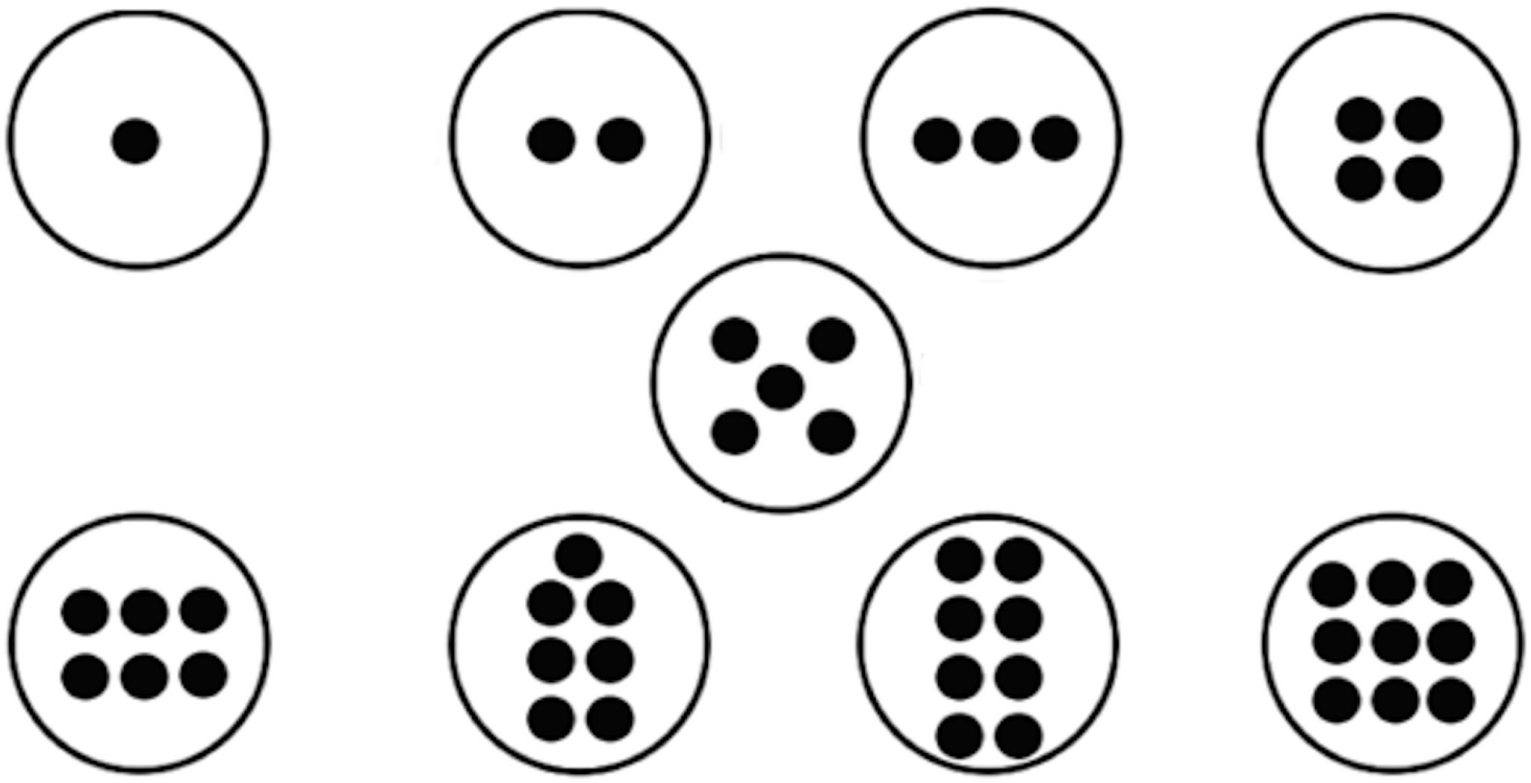
Dot matrix patterns used in Experiment 2.

The paradigm and stimuli as well as the timing of the presentation of the stimuli were the same as in Experiment 1 except that dot matrices were used instead of written number words in a 2x2x1 design. We chose to employ canonical dot matrices to aid in ease of recognition. The patterns used can be found in Fig 6.

It is important to note that the 600 ms (500 ms presentation + 100 ms gap) between the time S1 disappeared until S2 was presented in our experiments was most probably sufficient to encode S1. In their review of papers using dot matrices, Szűcs et al. [22] note that some investigators have used presentation times as short as 150 ms while others used long presentation times (e.g. 2000 ms or until response is given). Since comparison performance was the same with presentation times between 150 and 2000 ms using numbers larger than those in our experiments, it is reasonable to conclude that 600 ms was enough time for the numbers to translate into a central abstract representation, if indeed that is what were to occur.

### 3.3. Results of Experiment 2

#### 3.3.1. Effect of S1 on the RTs at S2/S3

**Fig 7:**
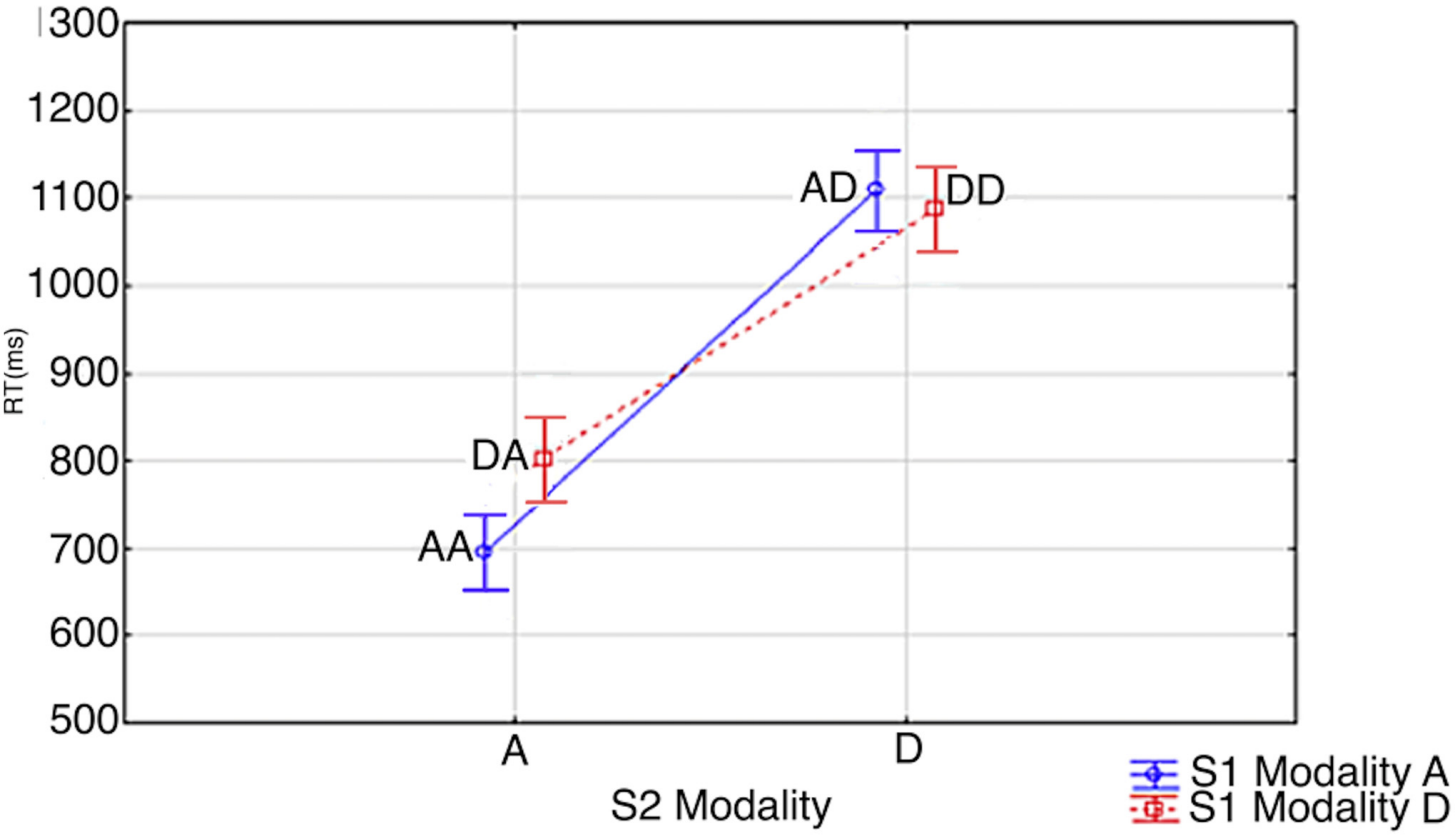
S1 Modality x S2 Modality. Vertical bars denote 0.95 confidence intervals for repeated measures ANOVA (Hollands & Jarmasz, 2009). S1 and S2 modality is signified by ‘A’ and ‘D’; ie. Arabic Digit (A) vs. Dot Matrix (D). As an example, AD signifies an Arabic digit at S1 and a dot matrix at S2.

RTs are shown in Fig 7. Data were assessed by a Correctness x S1 modality x S2 modality ANOVA. The .95 confidence intervals were computed for repeated-measures ANOVA (see Hollands and Jarmasz [32]). RTs were 42 ms faster when S1 was an Arabic digit than when it was a Dot Matrix (903 ms vs. 945 ms; S1 Modality: F(1,19) = 7.2177; p<0.0146; Effect size = 0.28; *Partial η* ^*2*^ = 0.71). RTs were 350 ms faster when S2 was an Arabic digit (749 ms vs. 1099; S2 Modality: F(1,19) = 485.34; p<0.00001; Effect size = 0.28; *Partial η* ^*2*^ = 0.71). Also, as in previous experiments, there was an S1 modality x S2 modality interaction (AA = 696 ms, DA = 802 ms, DD = 1087 ms, AD = 1110 ms; S1 Modality x S2 Modality: F(1,19) = 23.672, p<0.0001; Effect size = 0.55; *Partial η* ^*2*^ = 0.99) which showed that, when S2 was an Arabic digit, participants were quicker to verify the sum at S2/S3 when S1 was also a digit. There was no such effect when S2 was a dot matrix. According to S1 modality x S2 modality Tukey post hoc contrasts, there were significant differences between all conditions except between AD and DD (AD vs DD: p<0.2970; AA vs. AD: p<0.0002; AA vs. DA: p<0.0015; AA vs. DD: p<0.0002; AD vs. DA: p<0.0002; DA vs. DD: p<0.0002). There was no interaction between RTs and the correctness of the sum presented at S3 (925 ms vs. 922 ms; Correctness: *F*(1,19) = 0.0841, *p* = 0.7; Correctness x S1 Modality x S2 Modality: F(1,19) = 0.8875, *p* = 0.3).

#### 3.3.2. Effects on Accuracy

An assessment of accuracy based on the percentage of responses answered correctly was performed. A Correctness x S1 modality x S2 modality ANOVA showed no significant effects, including specifically no effects for the S1 modality (*p* = 0.11), S1 modality x S2 modality (*p* = 0.07), or the Correctness x S1 modality x S2 modality (*p* = 0.05). (See Table 1 for accuracy rate).

### 3.4. Discussion of Experiment 2

In experiment 2 the *NME* was replicated as S1 modality again affected RT when S2 was an Arabic digit. Similarly to when S2 was a written number word in previous experiments, there was no significant RT difference when S2 was a dot matrix. As explained more fully in the discussion for experiment 1, this makes sense if S1 retrieval and S2 encoding occur in parallel as the longer encoding time of dot matrices relative to Arabic digits would mask the effect.

Since dot matrices are purported as being a more abstract numerical surface form and since the triple-code and abstract modular models propose an abstract representation for number by default in memory, we adjusted the paradigm to show dot matrices instead of written number words in order to observe how this would affect the *NME*. In general, the results from the dot matrices were very similar to that of the written number words. There was no special RT advantage observed from presenting numbers as canonical dot matrices.

Similarly to the potential confound, due to the fixed modality of S2, in the Szűcs and Csépe [30] experiment which we controlled for in experiments 1 and 2, another potential confound to be examined concerns the fixed modality of S3 (the proposed result). In the condition in which S2 was a digit, in experiments 1 and 2, it is possible that S1 was processed more quickly when it was also a digit because S3 was always presented as an Arabic digit. Having S3 always appear as a digit could have prompted participants to process Arabic digits preferably, biasing the results and resulting in faster processing of Arabic digits due to strategic expectations. We addressed this issue in experiment 3.

## Experiment 3

### 4.1. Hypothesis of Experiment 3

Experiment 3 utilized the same paradigm as experiment 1, except that the number at S3 also was presented randomly as either an Arabic digit or a written number word rather than always being presented as a digit. If S3 always being presented as an Arabic digit was a major factor in preferential processing of Arabic digits in previous experiments, then a more balanced presentation of S3 varying both digits and written number words should result in the disappearance of the *NME* (Arabic digit retrieval advantage in Experiment 1).

### 4.2. Methods of Experiment 3

#### 4.2.1. Subjects

Seventeen subjects from experiment 2 participated in this experiment, ten of which were female. They were between the ages of 18 and 35 (median age: 23) and were from the Cambridge, UK area. Six of these also had participated in Experiment 1. Fourteen of the participants were students or former students from the University of Cambridge with the others being residents from the Cambridge area. Ten of the students were engaged in or had completed graduate studies and the rest were undergraduates. All reported themselves as being unimpaired in regards to math ability.

#### 4.2.2. Procedures

The paradigm and stimuli as well as the timing of the presentation of the stimuli were the same as in Experiment 1 except that, instead of a 2x2x1 design we used a 2x2x2 with S3 also being randomly presented as either an Arabic digit or a written number word.

### 4.3. Results of Experiment 3

#### 4.3.1. Effect of the modality of S1 and S2 on the RTs

**Fig 8:**
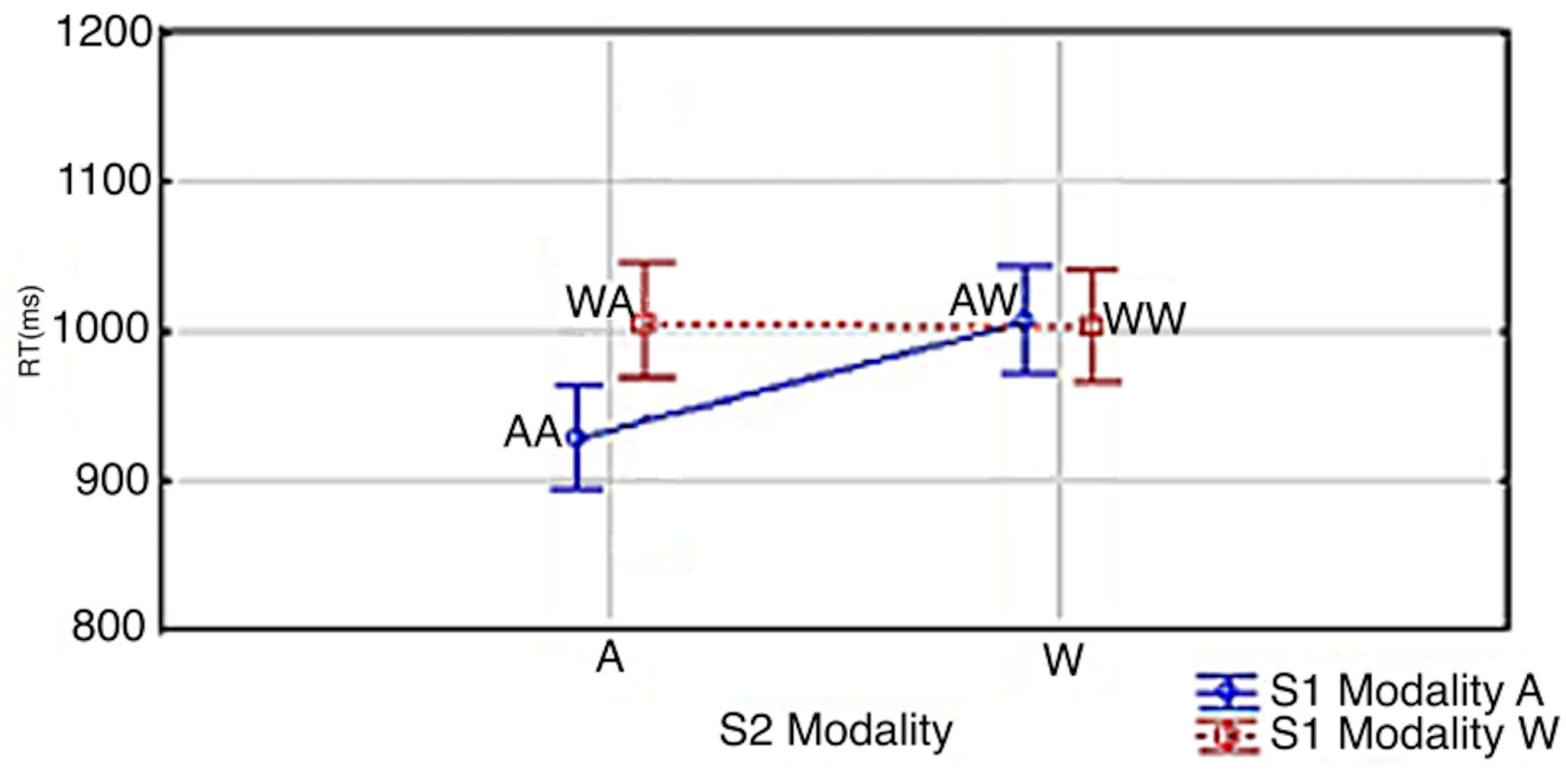
S1 Modality x S2 Modality. Vertical bars denote 0.95 confidence intervals for repeated measures ANOVA (Hollands & Jarmasz, 2009). S1 and S2 modality is signified by ‘A’ and ‘W’; ie. Arabic Digit (A) vs. Written Number Word (W). As an example, AW signifies an Arabic digit at S1 and a written number word at S2.

**Fig 9:**
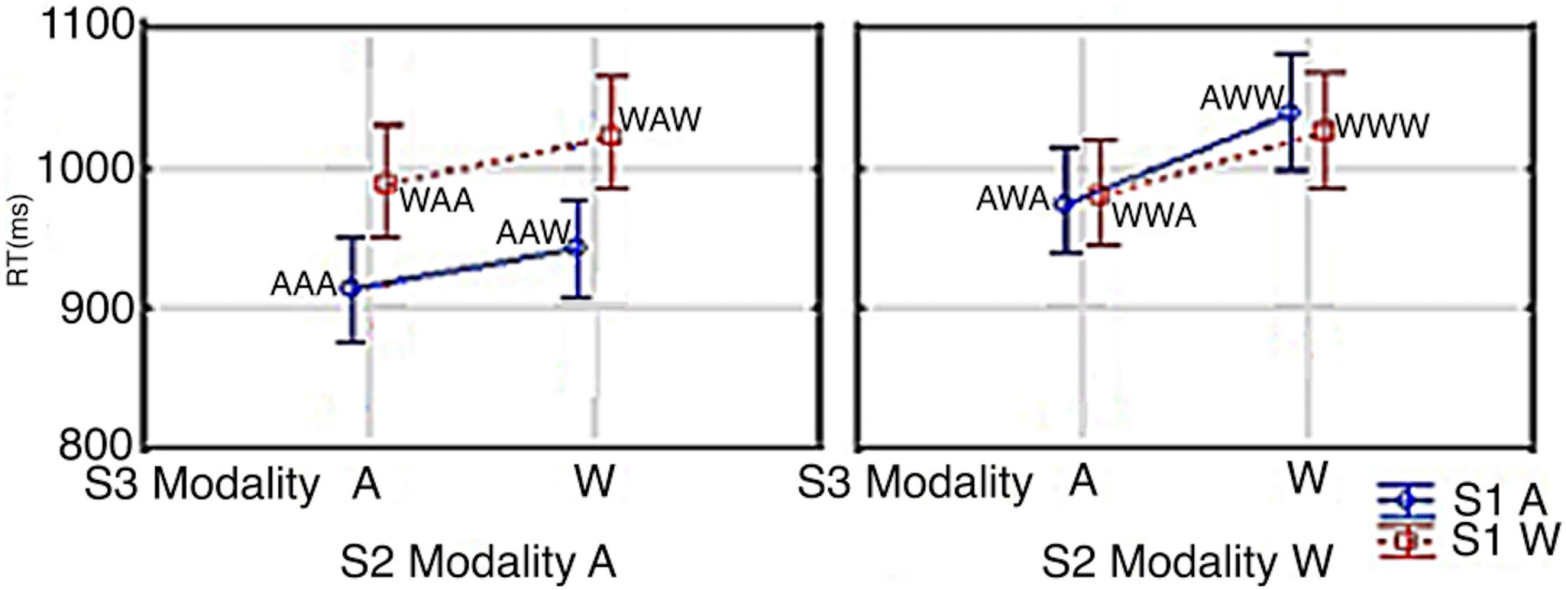
S3 Modality x S1 Modality x S1 Modality. Vertical bars denote 0.95 confidence intervals for repeated measures ANOVA (Hollands & Jarmasz, 2009). S1, S2 & S3 modality is signified by ‘A’ and ‘W’; ie. Arabic Digit (A) vs. Written Number Word (W). As an example, WAW signifies a written number word at S1 and S3 and an Arabic digit at S2.

RTs are shown in Figs 8 and 9. Data were assessed by a Correctness x S3 modality x S1 modality x S2 modality ANOVA. The .95 confidence intervals were computed for repeated-measures ANOVA (see Hollands and Jarmasz [32]). RTs at S2/S3 were 37 ms faster when S1 was an Arabic digit than when it was a number word (967 ms vs. 1004 ms; S1 Modality: *F*(1,16) = 19.229; *p*<0.0005; *Partial η* ^*2*^ = 0.65). Crucially, as in Experiment 1, there was an expected S1 modality x S2 modality interaction (S1 Modality x S2 Modality: *F*(1,16) = 29.183, *p*<0.0001; *Partial η* ^*2*^ = 0.65). The interaction resulted in shorter solution times when S2 was presented as an Arabic digit. This effect was modulated by S1 modality: RTs were the fastest when S1 was an Arabic digit (AA = 928 ms, WW = 1002 ms, WA = 1005 ms, AW = 1005 ms). A Tukey post hoc analysis showed congruency effects only between AA-WA, AA-AW, and AA-WW (AW vs WW: *p* = 0.9999; AW vs. WA: *p* = 0.9454; WA vs. WW: *p* = 0.9912; AA vs. AW: *p*<0.0002; AA vs. WA: *p*<0.0005; AA vs. WW: *p*<0.0002). Also, as in our previous experiments, the presentation of the answer at S3 as being either correct or incorrect did not show an interaction with how the modalities at S3, S1 or S2 correlated with the speed of the responses (Correctness x S3 modality x S1 modality x S2 modality: *F*(1,16) = 0.9772, *p* = 0.3).

#### 4.3.2. Effect of the modality of S3 on the RTs

Fig 9 shows RTs by S1 x S2 x S3. In all conditions, the answer at S3 was verified 108 ms faster when S3 was an Arabic digit (1039 ms vs. 1147 ms; S3 Modality: *F*(1,16) = 76.484; *p*<0.0001; *Partial η* ^*2*^ = 0.65). In trials in which S3 was an Arabic digit, RTs were 26 ms faster when S2 was an Arabic digit; and in trials in which S3 was a number word, RTs were 49 ms faster when S2 was an Arabic digit showing seemingly an additive effect of the time it takes to read/process an Arabic digit vs a written number word (AA = 951 ms, WA = 977 ms, AW = 982 ms, WW = 1031 ms; S3 Modality x S2 Modality: *F*(1,16) = 6.9276; *p*<0.0181; *Partial η* ^*2*^ = 0.24). Another observation from this experiment was that, in contrast to Experiment 1, there was no longer any RT difference between WA and AW/WW (see Fig 5 above).

#### 4.3.3. Effect of the previous trial’s S3 on the RTs at S2/S3

We considered the modality of the S3s that occurred immediately before the trial of the RT in question (previous S3s). There was no interaction. The RTs when the previous trial’s S3 was an Arabic digit was only 10 ms faster than when the previous trial’s S3 was a Number Word (989 ms vs. 979 ms; S3 Modality: *F*(1,16) = 2.7468; *p* = 0.11). An ANOVA of the modality of the previous S3 by the RTs at S2 also showed no significant effect (AA = 961ms, WA = 971ms, AW = 997ms, WW = 1007ms; Previous S3 x S2 Modality: *F*(1,16) = 0.00267; *p*>0.9).

#### 4.3.4. Effects on accuracy

As in Experiment 1, accuracy was assessed. A Correctness x Previous S3 modality x S1 modality x S2 modality ANOVA showed no significant effects, including specifically no effects for the Previous S3 modality (*p* = 0.314), the Previous S3 modality x S2 modality (F(*p* = 0.343), or the Correctness x Previous S3 modality x S2 modality (*p* = 0.1). A Correctness x Same S3 modality x S1 modality x S2 modality also showed no significant effects, including specifically no effects for the Same S3 modality (*p* = 0.0560), the Same S3 modality x S2 modality (*p* = 0.6), or the Correctness x Same S3 modality x S2 modality (*p* = 0.8) (See Table 1 for accuracy rate).

### 4.4. Discussion of Experiment 3

The *NME* was again replicated in Experiment 3: When S2 was an Arabic digit, RTs at S2/S3 were the fastest when S1 was also an Arabic digit. Also, as in Experiment 1, this effect was not shown when S2 was a written number word. However, unlike in Experiment 1 there was no longer any RT difference between the WA condition and the AW/WW conditions. This difference is independent of the *NME* and is likely due to the randomization of S3 which was implemented in order to control for the possibility that Arabic digits were preferred because they occurred more often throughout the experiment. The fact that the slight advantage of the WA condition relative to the AW and WW conditions disappeared in Experiment 3 suggests that there was indeed some preferential processing for Arabic digits in Experiment 1. However, this preferential processing only had a major impact on the encoding speed of S2, not on the retrieval speed of S1. The *NME* (AA vs. AW difference) was 93 ms in experiment 1 and 77 ms in experiment 3. The advantage of the WA condition relative to conditions AW and WW was 49 and 74 ms in experiment 1. This later advantage practically became zero in experiment 3. However, any potential reduction of the *NME* in experiment 2 definitely did not reach this magnitude relative to the earlier effect size of 93 ms (the reduction was at most 93–77 = 16 ms). Hence, it seems reasonable to assume that manipulating S3 did not have an effect or did not have a major effect on the *NME* at all.

## General Discussion

In this study, we set out to explore whether numbers are retrieved from a central amodal source in working memory or from separate notation-dependent representations. In our experiments, participants first saw an addend (S1) which disappeared and was followed by another addend (S2) and a proposed result (S3) which appeared simultaneously. Due to the design of this paradigm, participants were compelled to retrieve S1 from memory in order to add it to S2. Predictions in regards to what would occur when the participants retrieved S1 could be drawn from the three math cognition models which were summarized in the introduction. If the *abstract modular* model were correct, then when the participants were presented with S1 as either an Arabic digit or a written number word, for example, numbers would have been stored in working memory by translating them into a central abstract representation. If this were the case, then in all the trials in which S2 was an Arabic digit, the participants would have retrieved a number from the same central representation area in each trial in order to always add it to an Arabic digit representation. Since the modalities of the numbers in this scenario would always be the same, there should be no significant difference in RTs which are dependent on the original modality of S1. Similarly, in the most common version of the *triple code* model, the magnitude coding area of the brain would have acted as a central abstract representation area for numbers. In others words, the other two notation-dependent codes would have necessarily been translated through the abstract magnitude code for any task in which the comprehension of number was required. This being the case, the predictions for the *triple code* model would be the same as for the above *abstract modular* model. Conversely, if the *encoding-complex* model (or an encoding-complex version of the *triple-code* model) were correct, the numbers would have been stored and retrieved from notation-dependent representations without a need for any central abstract representation area. Therefore, this model predicted differences in RT which would be dependent on the modality of S1.

In response to our main question, in three independent experiments we have shown the *numeral modality effect* (*NME*), namely that the RTs at S2/S3 were dependent on the modality of S1 in the conditions in which S2 was an Arabic digit. This suggests that separate notation-dependent representations of number (not a central source) may be the correct model. The *NME* was not observed when S2 was a written number word or a dot matrix. We reason that this can best be explained by assuming that the retrieval of S1 and the encoding of S2 occur in parallel. Since the written number words and dot matrices take longer to encode (as shown in our data), it is likely that the longer processing time is masking the *NME* (see discussion of experiment 1 for a fuller explanation). The possibility that S1, S2, and even perhaps S3, were retrieved and encoded in parallel rather than sequentially may be important to take into account in similar experiments. Also of import to note is that the results of this study should not be construed to mean that there is no area in the brain responsible for the abstract representation of number, but rather they argue against any representation being the central “default” representation for number.

## Conclusion

One of the key questions in math cognition concerns whether numbers are necessarily stored into and retrieved from a central abstract representation of quantity or as notation-dependent representations. The findings replicated in three experiments seem to indicate that numbers are retrieved from distinct sources, and thus seem to argue for notation-dependent representations. Due to its sensitivity to number modality, the *NME* that is explored here could serve to be a valuable tool as we strive to understand number cognition.
